# Obesity and Diabetes in Jordan: Findings From the Behavioral Risk Factor Surveillance System, 2004

**Published:** 2007-12-15

**Authors:** Henry Walke, Ali H Mokdad, Meyasser Zindah, Adel Belbeisi

**Affiliations:** Division of Epidemiology and Surveillance Capacity Development, Coordinating Office of Global Health, Centers for Disease Control and Prevention; Behavioral Surveillance Branch and Director, Behavioral Risk Factors Surveillance System, National Center for Chronic Disease Prevention and Health Promotion, Centers for Disease Control and Prevention, Atlanta, Georgia; Noncommunicable Disease Department, Jordan Ministry of Health, Amman, Jordan; Jordan Ministry of Health, Amman, Jordan

## Abstract

**Introduction:**

Chronic diseases are the leading cause of morbidity and mortality in Jordan. The Jordanian Ministry of Health, in collaboration with the Centers for Disease Control and Prevention, established a behavioral risk factor surveillance system to monitor the behavioral risk factors associated with chronic diseases.

**Methods:**

We used a multistage sampling design to select households from which we then randomly selected and interviewed one adult aged 18 years or older. A random subsample of the adults interviewed were then invited to visit the local health clinic, where we obtained medical measurements, including blood lipids (low-density lipoprotein, high-density lipoprotein, and triglycerides) and fasting blood glucose.

**Results:**

Approximately 9% of the participants in the subsample who underwent medical testing reported having been diagnosed with diabetes previously, compared with 16.9% diagnosed in our laboratory testing. About 12.3% of the participants were glucose intolerant, and about 35% were obese. Obesity was significantly associated with diabetes, high blood pressure, high cholesterol, and asthma. Compared with adults of normal weight, obese adults had an adjusted odds ratio of 3.27 (95% CI, 1.58–6.76) for diabetes, 3.69 (95% CI, 2.13–6.39) for high blood pressure, 3.45 (95% CI, 1.68–7.10) for high cholesterol, and 5.12 (95% CI, 1.53–17.19) for asthma.

**Discussion:**

Obesity, poor diet, and physical inactivity create a major chronic disease burden in Jordan that is likely to increase substantially in the next few years. Our findings argue for establishment of a more preventive orientation in health care and public health systems in Jordan.

## Introduction

Chronic diseases are the leading cause of mortality in Jordan. According to Jordan Ministry of Health (MOH) mortality statistics, 38.2% of deaths in 2003 were attributed to cardiovascular diseases and 14.3% to cancers (unpublished raw data). The Jordan MOH, with assistance from the Centers for Disease Control and Prevention and the World Health Organization, established a behavioral surveillance program to monitor the prevalence of risk factors associated with these diseases. The first Behavioral Risk Factor Survey in Jordan, which was conducted in 2002, revealed that self-reported chronic disease risk factors are highly prevalent. Among adults aged 18 years or older, the self-reported diagnosed prevalence of obesity was 12.8%; diabetes, 6.4%; high blood pressure, 22.2%; high blood cholesterol, 20.9%; and asthma, 5.1% (1). By contrast, a household survey that the Jordan MOH conducted in 1996 of adults aged 25 or older measured key chronic disease indicators (2). At that time, the measured prevalence of obesity was 30.8%; diabetes, 6.8%; high blood pressure, 31.8%; and high blood cholesterol, 9.1%.

## Methods

In our analysis we used data from the Behavioral Risk Factor Surveillance System, a nationally representative survey of Jordanian adults aged 18 years or older that the Jordan MOH conducted in 2004 (3). This survey, which included data from medical examinations, reported on changes over time in obesity and diabetes, on the percentage of the population with undiagnosed chronic diseases, and on the relationship between body mass index and selected chronic disease risk factors.

In its 2004 survey, the Jordan MOH used a multistage sampling design to select the households in which the survey was administered. The master sampling frame of census enumeration blocks from the 1994 Jordan Census was used to select the sample of blocks, or primary sampling areas, from which households were selected. This sampling frame was stratified by governorate, major city, other urban area, and rural area into 29 strata that fit within three regions, North, Middle, and South. Geographic ordering of the blocks in the frame provided implicit stratification. Within each stratum the sample blocks were selected systematically with probability proportional to size. Eight households were selected from each block. In each house one adult aged 18 years or older was randomly selected and interviewed in person in Arabic.

Interviews were conducted between October 1 and December 13, 2004. In the total 3520 households selected, 3334 adults were interviewed, a response rate of 94.7%. The survey instrument included questions on demographics (e.g., age, sex, educational status), health status, health care access, hypertension awareness, cholesterol awareness, diabetes, asthma, heart disease, tobacco use, physical activity, nutrition, weight, height, oral health, eyesight, women's health, and use of medical services. All questions were translated from English into Arabic and then back-translated to ensure accuracy. The survey was pilot tested before implementation.

To compare self-reported health information to actual medical measurements, we randomly selected a subsample of 833 survey respondents from the 3334 people interviewed and invited them to participate in a medical examination. Each of the 833 was evaluated at a local health center where blood pressure, weight, height, and waist circumference were obtained. A fasting blood sample was obtained from each (participants fasted from midnight preceding their clinic visit) and sent to a central laboratory where total cholesterol, high-density lipoprotein cholesterol (HDL-C), low-density lipoprotein cholesterol (LDL-C), triglycerides, and fasting blood glucose were measured. We provided standardized training to the attending physicians of the participating local health clinics, and all participating physicians used the same standard equipment for blood testing and for measuring height and weight. A total of 710 of the 833 survey respondents participated in the medical evaluation for a response rate of 85.2%. Participants completed a consent form, and the study design was approved by the Jordan MOH.

Self-reported data on participants in the medical evaluation were obtained from survey questions. Weight and height were assessed by asking, "About how much do you weigh?" and "About how tall are you?" We then used these data to calculate body mass index (BMI [weight in kg/height in m^2^]). Participants were classified as normal weight if their BMI was less than 25, overweight if their BMI was from 25 to 29, and obese if their BMI was 30 or greater. To assess diabetes prevalence, we asked, "Have you ever been told by a health professional that you have diabetes?" The type of diabetes was not assessed. We assessed high blood pressure by asking, "Have you ever been told by a health professional that you have high blood pressure?" For high cholesterol we asked, "Have you ever been told by a health professional that your blood cholesterol is high?" To assess health status, we asked, "Would you say that, in general, your health is excellent, very good, good, fair, or poor?" Participation in vigorous physical activity was assessed with the question, "On average, how many days a week do you get at least 20 minutes of vigorous physical activity? Include your time at work." For moderate physical activity we asked, "On average, how many days a week do you get at least 30 minutes of moderate physical activity? Include your time at work." To assess consumption of fruits and vegetables we asked, "How many cups of fresh or cooked vegetables did you have yesterday?" and "How many cups of fruits or fresh juices did you have yesterday?"

We defined high cholesterol as having a cholesterol level of 240 mg/dl or greater or being on high cholesterol medication; high blood pressure as 140/90 mm Hg or greater or being on high blood pressure medication; impaired fasting glucose as from 100 mg/dl to 125 mg/dl, and diabetes as 126 mg/dl or greater or being on insulin or oral hypoglycemic medication. We limited our analyses to participants with laboratory data.

We used logistic regression analysis to estimate odds ratios of health risk factors associated with obesity. All analyses were adjusted for sex, age, education, smoking, fruit and vegetable consumption, physical activity, high blood pressure, high cholesterol level, and diabetes. For all analyses, *P* values <.05 were considered statistically significant. We used SUDAAN statistical software (Research Triangle Institute, Research Triangle Park, North Carolina) in all analyses to accommodate the complex survey sampling design.

## Results

Interviewees who agreed to undergo the medical examination were more likely to be women, older, and with lower educational levels (Table 1). They also were more likely to have been diagnosed with high blood pressure, high cholesterol, or diabetes; however, differences in these measurements were not statistically significant.

The percentage of participants in the medical evaluation with undiagnosed diabetes, high blood pressure levels, or high cholesterol levels was very high (Table 2). The total prevalence of obesity based on measured weights and heights was 34.8% compared with 22.4% based on self-reported data. Nine percent of participants reported that they had been diagnosed with diabetes compared with 16.9% diagnosed by laboratory testing. In addition, about 12.3% of the participants had impaired fasting glucose (not shown in Table 2).

Obesity was significantly associated with diabetes, high blood pressure, high cholesterol, and asthma (Table 3). Compared with adults of normal weight, obese adults had a fully adjusted odds ratio (OR) of 3.27 (95% confidence interval [CI], 1.58–6.76) for diabetes; an OR of 3.69 (95% CI, 2.13–6.39) for high blood pressure; an OR of 3.45 (95% CI, 1.68–7.10) for high cholesterol; and an OR of 5.12 (95% CI, 1.53–7.19) for asthma.

## Discussion

Our study revealed a high prevalence of obesity and diabetes among Jordanian adults that is similar to that among adults in the United States, who in 2005 had a prevalence of 23.9% for obesity (4) and 7.3% for diabetes (5). Moreover, our data revealed that a high percentage of people with diabetes are not diagnosed. The high prevalence of diabetes and obesity coupled with high levels of undiagnosed conditions point to the need for immediate implementation of programs to prevent and control the burden of chronic diseases in Jordan.

The high rates of undiagnosed diabetes, high blood pressure, and high cholesterol will result in severe chronic disease complications. Prevention and early detection are necessary to prevent these diseases and their consequences. Early diagnosis likely will prompt individuals to improve their health behaviors.

Jordan has witnessed a fast rise in risk factors for chronic diseases. The diagnosed prevalence of diabetes among Jordanian adults aged 18 years or older increased from 6.4% in 2002 to 7.5% in 2004 (1). During the same period, the self-reported prevalence of obesity increased from 12.8% to 19.5%, a 50% increase in 2 years. (Compared with data from the Prevalence of Risk Factors on Noncommunicable Diseases, Jordan 1996, Jordan's 1996 national household survey for adults aged 25 or older, the comparable percentages in 2004  increased from 30.8% to 37.8% for obesity and from 6.8% to 17.9% for diabetes [2].) Such large increases are not unprecedented. The self-reported prevalence of obesity in the state of Georgia in the United States increased from 11.5% in 1996 to 19.2% in 1998, a 67% increase (6). In the total U.S. population, obesity prevalence increased from 12.0% to 17.9% between 1991 and 1998, an increase of 49.2% (7).

These rapid increases in obesity and diabetes reflect a major shift in behaviors associated with chronic diseases. In the 2004 Behavioral Risk Factor Survey for Jordan, about 50% of Jordanian adults reported that they did not engage in any physical activity (3). Perhaps the most striking finding from this survey was the low intake of fruits and vegetables among participants: only about 19% of survey respondents reported having consumed three or more cups of fruits, fresh juices, or vegetables the previous day. In addition, awareness of what constituted a healthy weight was poor: 27.8% of obese Jordanians (BMI ≥30) reported that they considered their weight to be about average. In contrast, only 11.9% of obese males and 4.6% of obese females in the United States reported that they were of normal weight or underweight (8).

The burden of diabetes in Jordan is very high. In 2004, 62.9% of people with diabetes reported that they had never had their feet checked for sores or irritations, and 45.3% had not had an eye examination in the past 12 months (3). Among people with diabetes who reported ever having had an eye examination, 36.3% were told they had eye complications. This finding may reflect late diagnosis of the disease.

A particularly disturbing finding from the Jordan's 2004 Behavioral Risk Factor Survey was that only 23.3% of obese survey participants who had visited a health care facility in the past 6 months had been advised by a health professional to lose weight. In clinical settings, overweight and obesity should be assessed as risk factors for chronic diseases. Indeed, people who receive advice from a health professional to lose weight are more likely to attempt to do so than those who do not receive this advice (9). Health professionals should assess body weight and recommend weight loss (a combination of a low-calorie diet and increased physical activity) to overweight and obese patients and weight maintenance to patients of normal weight.

Although overweight and obese people need to reduce caloric intake and increase physical activity, other factors play a role in helping them to lose weight and to prevent or control diabetes. In addition to the role of health care providers in counseling their overweight and obese patients about the importance of achieving and maintaining a healthy weight, workplaces should provide opportunities for employees to be physically active onsite. Schools should offer physical education that encourages lifelong physical activity, and parents should reduce their children's television and computer time and encourage active play. Urban policy makers should work toward providing an environment that enables and encourages individuals to be physically active. In general, restoring physical activity to daily routines is crucial.

Unfortunately, the prevalence of obesity and diabetes has increased in Jordan since 1996 and most likely will continue to rise in the years ahead unless effective interventions are implemented. In the past 25 years, several promising approaches have been identified for clinical and public health action. To reduce the risk of obesity and diabetes, it is crucial to begin now to implement multicomponent interventions for weight control, healthy eating, and physical activity.

Our survey has some limitations. Its study design is cross-sectional, and the survey was not conducted throughout the year. Seasonal behaviors related to dietary intake (e.g. variations related to seasonal availability of fruits) may not be representative, and cause and effect cannot be determined for the associations between BMI and selected health conditions. In addition, although we selected the subsample of survey respondents randomly for medical evaluation from the pool of people participating in the household interview, self-selection bias may have been a factor in the differences noted between the group undergoing medical examination and the larger group that participated in the household interview from which the subsample was selected.  Our small sample size in the medical examination group did not allow us to examine the relationships among chronic disease risk factors by demographics and socioeconomic status, and thus important differences between BMI and selected risk factors, for example, across age or income categories, may have been missed. Finally, although our survey questions related to behavioral risk factors have documented validity in English, the questions were translated into Arabic; there have not been studies to demonstrate the validity of such questions in the Arabic-speaking world (10).

In summary, obesity, poor diet, and physical inactivity create a major chronic disease burden in Jordan. The rapid increase in the prevalence of obesity and diabetes indicate that this burden is likely to grow substantially in the next few years. The problem is compounded by an increasing life expectancy and, thus, an older population at a time when Jordan struggles to accommodate rising costs of health care. Our findings argue for a stronger emphasis on prevention in health care and public health systems in Jordan.

## Figures and Tables

**Table 1:** Participant Characteristics, Household Interview and Medical Examination, Behavioral Risk Factor Surveillance System, Jordan, 2004

Characteristic	All Participants N = 3342% (SE^a^)	Participants in Household Interview n = 2632% (SE^a^)	Participants in Medical Examination n = 710% (SE^a^)
**Sex**			
Men	40.3 (0.69)	42.3 (0.88)	32.5 (2.30)
Women	59.7 (0.69)	57.7 (0.88)	67.5 (2.30)
**Age, y^b^**			
18-34	41.3 (1.36)	42.9 (1.53)	35.7 (1.95)
35-49	32.0 (0.90)	31.3 (1.11)	34.5 (1.53)
50-64	17.5 (-0.82)	17.0 (0.93)	19.3 (1.09)
≥65	9.2 (0.71)	8.9 (0.70)	10.5 (1.29)
**Education**			
Never attended school	14.7 (0.75)	14.1 (0.48)	17.2 (1.46)
Primary school	28.0 (0.90)	27.2 (0.92)	31.2 (1.80)
Secondary or technical school^c^	44.3 (0.95)	44.5 (1.03)	43.2 (1.77)
University or more^b^	13.0 (1.09)	14.2 (1.28)	8.4 (1.09)
**Current smoker^d^**	22.8 (0.78)	23.6 (0.78)	20.0 (1.63)
**Obese^e^**	19.5 (0.91)	18.7 (0.93)	22.4 (2.09)
**Engages in moderate activity^f^**	48.2 (0.89)	45.9 (2.32)	57.4 (2.19)
**Has been told has**			
High blood pressure	14.8 (0.69)	14.8 (0.73)	15.2 (1.52)
High blood cholesterol	6.4 (0.57)	5.8 (0.51)	8.5 (1.28)
Diabetes	7.5 (0.58)	7.1 (0.60)	9.0 (1.16)

a Standard error.

b Some categories do not total 100% because of rounding.

c Attended or graduated.

d Ever smoked ≥100 cigarettes in a lifetime and currently smoke every day or some days.

e Defined as a body mass index (BMI) ≥30.

f Any moderate activity (i.e., activity resulting in light sweating, small increases in breathing or heart rate).

**Table 2:** Chronic Disease Risk Factors Among Participants in Medical Examination, by Selected Demographic Characteristics, Behavioral Risk Factor Surveillance System, Jordan, 2004

	Sex		Age Groups				Total% (SE^a^)

	Male% (SE^a^)	Female% (SE^a^)	18–34% (SE^a^)	35–49% (SE^a^)	50–64% (SE)	≥65% (SE^a^)	
**High blood pressure**							
Self-reported	14.5 (2.41)	15.5 (1.62)	2.5 (0.95)	11.3 (1.87)	35.9 (4.05)	34.1 (6.82)	15.2 (1.52)
Measured	36.3 (3.59)	27.3 (1.98)	9.4 (2.30)	28.3 (3.53)	55.2 (3.78)	61.4 (5.52)	30.2 (1.83)
**High cholesterol**							
Self-reported	11.8 (1.90)	7.0 (1.37)	0.9 (0.58)	8.5 (1.75)	18.6 (3.55)	18.2 (4.3)	8.5 (1.28)
Measured	25.6 (3.42)	21.9 (2.07)	8.0 (1.30)	25.5 (3.04)	38.0 (4.08)	39.2 (6.51)	23.1 (1.71)
**Diabetes**							
Self-reported	9.8 (1.95)	8.6 (1.36)	0.81 (0.60)	6.0 (1.69)	22.3 (3.56)	22.8 (4.8)	9.0 (1.16)
Measured	17.7 (2.38)	16.5 (1.38)	6.1 (1.56)	12.7 (1.95)	32.3 (3.72)	39.0 (6.65)	16.9 (1.24)
**Obesity**							
Self-reported	16.7 (2.33)	25.7 (2.48)	13.0 (2.45)	28.8 (3.54)	25.5 (3.97)	28.7 (6.77)	22.4 (2.09)
Measured	21.1 (2.81)	41.5 (2.66)	19.1 (2.80)	42.7 (2.62)	45.3 (4.58)	43.4 (6.72)	34.8 (2.37)

a Standard error.

**Table 3:** Relationship Between Body Mass Index and Selected Health Conditions, Behavioral Risk Factor Surveillance System, Jordan, 2004)^a^

Health Condition	Body Mass Index (BMI)^b^		

	Normal BMI <25	Overweight BMI 25–29	Obese BMI ≥30
**Diabetes^c^**			
Age-adjusted OR	1.00	2.03 (95% CI, 0.95-4.35)	3.84 (95% CI, 1.89-7.78)
Fully adjusted OR	1.00	2.17 (95% CI, 1.07-4.39)	3.27 (95% CI, 1.58-6.76)
**High blood pressure^c^**			
Age-adjusted OR	1.00	1.23 (95% CI, 0.78-1.95)	3.33 (95% CI, 1.93-5.73)
Fully adjusted OR	1.00	1.24 (95% CI, 0.74-2.08)	3.69 (95% CI, 2.13-6.39)
**High cholesterol^c^**			
Age-adjusted OR	1.00	2.97 (95% CI, 1.52-5.81)	3.45 (95% CI, 1.65-7.22)
Fully adjusted OR	1.00	3.17 (95% CI, 1.59-6.34)	3.45 (95% CI, 1.68-7.10)
**Asthma^d^**			
Age-adjusted OR	1.00	1.18 (95% CI, 0.37-3.78)	4.18 (95% CI, 1.55-11.24)
Fully adjusted OR	1.00	1.28 (95% CI, 0.37-4.43)	5.12 (95% CI, 1.53-17.19)
**Fair or poor health^d^**			
Age-adjusted OR	1.00	1.45 (95% CI, 0.75-2.81)	1.61 95% CI, (0.87-2.96)
Fully adjusted OR	1.00	1.34 (95% CI, 0.72-2.48)	1.33 (95% CI, 0.71-2.49)

OR indicates odds ratio; CI, confidence interval.

a Fully adjusted data are adjusted for sex, age, education, smoking, fruit and vegetable consumption, physical activity, and two or more of the following: high blood pressure, high cholesterol level, and diabetes.

b Weight in kg/height in m^2^

c Measured.

d Self-reported.
